# Shared Decision Making for Radioiodine Therapy and the Actual Pattern of Care in Intermediate-Risk Differentiated Thyroid Carcinoma

**DOI:** 10.3389/fnume.2021.797522

**Published:** 2022-01-21

**Authors:** Friederike Eilsberger, Markus Luster, Christoph Reiners

**Affiliations:** ^1^Department of Nuclear Medicine, University Hospital Marburg, Marburg, Germany; ^2^Department of Nuclear Medicine, University Hospital Würzburg, Würzburg, Germany

**Keywords:** shared-decision-making, intermediate-risk, thyroid cancer, radioiodine therapy, RIT

## Abstract

Radioiodine therapy (RAI) is usually a standard procedure performed after thyroidectomy in differentiated thyroid cancer (DTC). While the indication for RAI in high-risk patients has been established in various national and international guidelines, there is an ongoing discussion with regard to intermediate-risk patients. In addition to the inconsistent definition of this risk category, the absence of large multinational prospective randomized controlled trials forms the basis of the debate. In this context, the actual pattern of care and national guidelines in the country where the patient is living plays an important role with respect to regional iodine supply and goiter prevalence, preoperative diagnostics (fine needle aspiration biopsy), and corresponding surgical strategies. Participatory decision-making between physician and informed patient, which is demanded in principle today anyway, is of particular importance in this situation. This article will discuss the approach of shared decision making for radioiodine therapy in intermediate-risk DTC.

## Introduction

After thyroidectomy, radioiodine therapy (RAI) is the standard treatment for most patients with differentiated papillary or follicular thyroid cancer (DTC). Experts from different disciplines in various countries have divergent views on the indication of RAI in patients who are not classified as high-risk, whereby these classifications also differ. Also, the pattern of care standards in different countries are not necessarily the same, which is why approaches cannot be directly applied without restriction. The issue becomes even more complex by the lack of randomized prospective trials with evidence that provides a basis for recommendations. The absence of such evidence makes participatory decision making between physician and patient particularly important in this situation.

## Risk Classification

It is crucial to consider the divergent definition of intermediate-risk patients, as various national and international guidelines and societies including the European Thyroid Association (ETA) and the American Thyroid Association (ATA) use non-uniform risk classifications, whereby additionally a difference must be considered between the risk of DTC related death and the risk of recurrence.

Almost all risk categorization systems are predicated on the extent of disease, with tumors confined to the thyroid gland with no evidence of lymph node or distant metastases usually being classified as “low-risk.” “High-risk” tumors are generally defined in the presence of extensive local extrathyroidal invasion or metastatic disease. In the case of lymph node metastases, they may be more appropriately classified as “intermediate-risk.”

Guidelines and consensus reports by the ETA stratified in the past the patients regarding *mortality risk* following the TNM system into the groups of very low-risk, low-risk and high-risk (1, 2). This low-risk group corresponds most appropriately to an intermediate-risk group, which was absent from this classification under this designation. Patients with an unifocal carcinoma (≤ 1 cm) with no extension beyond the thyroid capsule and without local or distant metastases were classified as very low-risk. Patients with distant metastases, incomplete tumor resection or complete tumor resection but a high-risk for recurrence or mortality (T3/4 or lymph node metastases) were assigned to the high-risk group. All other patients were defined as low-risk patients.

This approach was particularly useful because the classification approximates routine clinical practice; moreover, it is the method most commonly used in Europe. However, in its latest consensus paper, the ETA adopts the ATA classification (3).

Regarding the ATA guideline, this classification refers to the *risk of recurrence* in a low-risk, intermediate-risk and high-risk group (Table 1) (4). Low-risk is for example stratified for papillary thyroid cancer without local or distant metastases, no macroscopically remaining tumor tissue, no tumor invasion of loco-regional tissues or structures, no aggressive histology, no radioiodine-avid metastatic foci outside the thyroid bed, no vascular invasion and clinical N0 or ≤ 5 N1 micrometastases. Classified as intermediate-risk are microscopic invasion of tumor in the perithyroidal soft tissues, iodine-avid metastatic foci in the neck, aggressive histology, papillary thyroid cancer with vascular invasion, clinical N1 or >5 pathologic N1 (<3 cm) or a multifocal papillary microcarcinoma with extrathyroidal extension and/or BRAF V600E mutation.

**Table 1:** Risk of recurrence according to the 2015 ATA guidelines (4).

Low-risk	- Papillary thyroid cancer with: No local or distant metastases No invasion in loco-regional tissues or structures No remaining macroscopic tumor tissue No aggressive histology No vascular invasion No RAI-avid metastatic foci outside the thyroid bed Clinical N0 or ≤ 5 N1 micrometastases (<0,2 cm) - Intrathyroidal, encapsulated follicular variant of papillary thyroid cancer - Intrathyroidal, well differentiated follicular thyroid cancer with capsular invasion and no or minimal (<4 foci) vascular invasion - Intrathyroidal, papillary microcarcinoma, unifocal or multifocal, including BRAFV600E mutated (if known)
Intermediate-risk	- Papillary thyroid cancer with vascular invasion - Aggressive histology - Microscopic invasion of tumor into the perithyroidal soft tissues - RAI-avid metastatic foci in the neck - Clinical N1 or >5 pathologic N1 (<3 cm) - Multifocal papillary microcarcinoma with extra thyroidal extension and BRAFV600E mutated (if known)
High-risk	- Macroscopic invasion of tumor into the perithyroidal soft tissues - Incomplete tumor resection - Distant metastases - Postoperative serum thyroglobulin suggestive of distant metastases - Pathologic N1 with any metastatic lymph node ≥3 cm - Follicular thyroid cancer with extensive vascular invasion (>4 foci)

As these descriptions show, different definitions of the intermediate group with respect to risk (mortality vs. recurrence) and staging (TNM system only vs. TNM + additional parameters) must be taken into account in the various classifications.

## Guideline Recommendations

The established aims of RAI are primarily to improve disease-specific survival, progression-free survival and to decrease recurrence rates by targeting occult lesions such as lymph node metastases or to treat known metastases (5). The indication of “prophylactic” ablation, in view of highly sensitive thyroglobulin assays and high-resolution sonography, may be considered secondary, but it inevitably results under adjuvant therapy. During RAI, whole-body images are performed in the sense of theranostics, which allow localization of pathological radioiodine uptake corresponding to tumor remnants, lymph node or distant metastases thus contributing – at least in patients with high-risk disease – to histological and clinical staging.

The current German guideline for radioiodine therapy of DTC, published in 2016, recommends RAI for patients with DTC >1 cm, thus low-risk (pT1b/2, cN0, pN0, M0) and high-risk (pT3/4, N1, M1) patients (6). In addition, for DTC <1 cm, the following factors should also be weighed in the decision for or against RAI (5): multifocality, capsule infiltration, infiltrative tumor growth, desmoplastic fibrosis, possibly BRAF V600E mutation, tumor diameter 6–10 mm, preoperative clinical carcinoma detection, a history of familiarity or percutaneous irradiation of the soft tissues of the neck. An updated multidisciplinary comprehensive guideline on thyroid carcinoma with possible changes in these recommendations is expected soon.

Similar to the German guideline is the recommendation of the European Association of Nuclear Medicine (EANM), published in 2008, which recommends RAI in all patients except in cases of DTC ≤ 1 cm without unfavorable histology, history of radiation exposure, evidence of metastasis and thyroid capsule invasion (7).

In the British guideline, published in 2014, RAI (8) is recommended for patients in the definite indication category pT3/4 and M1, personalized decision making is recommended in T1b/2 and N1 with regard to factors as resected metastatic lymph nodes of large size, multiple metastatic lymph nodes, unfavorable histological type, widely invasive histology, large tumor size, extrathyroidal extension, high ratio of positive to negative lymph nodes and extracapsular nodal involvement. No indication is seen in patients with papillary thyroid cancer ≤ 1 cm or minimally invasive follicular thyroid cancer without angioinvasion or invasion of thyroid capsule.

In the ATA guideline, published 2016, RAI is recommended for high-risk patients and “should be considered individually” in intermediate-risk patients (4).

In a previous ETA consensus paper, published 2005, (2) the authors described the reduction of the recurrence rate and a potential longer survival for high-risk patients after RAI, while no indication was described for very low-risk patients. There was no consensus for RAI in low-risk patients, because of uncertainties whether ^131^I should be generally offered or be selectively administered. The latest ETA consensus paper, published 2021, recommends that the decision to RAI in low-risk patients should be based on the presence of individual risk modifiers; in intermediate-risk patients, it states that RAI therapy may be indicated and should be should be adapted to the individual case (3).

In addition to official guidelines, there are also consensus statements from international groups composed of various societies. The most important example is the “Martinique Project,” in which experts from the ATA, EANM, SNMMI and ETA collaborated. The results of this 2019 working group were captured in the publication titled “Controversies, Consensus, and Collaboration in the Use of ^131^I Therapy in Differentiated Thyroid Cancer” (5).

In Principle 5, the authors state that optimal patient selection for adjuvant ^131^I treatment requires consideration and evaluation of multiple factors, circumstances beyond risk stratification and postoperative disease status. It is emphasized that there is most likely not one “right” way to treat DTC patients. The authors highlight that patient preferences and values, in addition to “traditional” factors such as postoperative risk assessment, the estimated likelihood that ^131^I administration will positively affect clinical outcomes of interest (disease-specific mortality, recurrence), and the assessment of potential side effects, are critical to decision making, as are factors such as the availability and quality of ultrasound and thyroglobulin assessment, the quality of surgery performed, and local disease management preferences are additional key elements to consider in assessing whether a patient might individually benefit from adjuvant ^131^I treatment. It is also critiqued that most of these factors were probably not adequately considered in the published retrospective studies.

The authors intensely emphasize the need to discuss and understand patient preferences and values and to incorporate them into shared decision making, as little high-quality prospective evidence is available and for many patients the decision for or against RAI therapy can be justified on the basis of the available literature. Thus, the decision of whether to proceed with RAI therapy is critically influenced by patients' wishes, fears, objections, and hopes.

The authors point out the duty of the treating physician to assess and evaluate the pros and cons of RAI therapy as objectively as possible in the individual situation of the patient in the individual health care system.

This overview of the selected guidelines presented (Table 2) illustrates the spectrum of recommendations for RAI in intermediate-risk patients. However, it is important to note, that the guidelines and recommendations of individual countries are not directly transferable, as also addressed by the Martinique group (5). Especially with regard to the ATA guideline, for example a transfer to a German setting remains difficult due to various factors. Among other aspects still existing iodine deficiency in Germany is followed by a high incidence of endemically enlarged thyroid glands with multiple focal lesions (9, 10). DTC cases are more likely to be detected as incidental findings during supposedly benign thyroid surgery (11). A significant role in the differences in surgical approach is played by the fact that preoperative suspicion of thyroid carcinoma is present in only 37% of German patients but 84% of American patients (12). In a recent study from Marburg, only 19% of 142 DTC patients had a fine needle aspiration before surgery (13). Furthermore, the Marburg study revealed the finding that frozen section histology was performed intraoperatively in only 56% of patients (13). On the other hand, this explains the fact that in Germany a DTC in more than 50% of cases is an unexpected incidental finding that requires follow-up surgery. In the USA, by contrast, the number of thyroid surgeries in 2006 was 93.000 (corresponding to 31/100.000 population). Thyroid operations were thus performed about 3 times less frequently in USA - in relation to the population - than in Germany (14). Thyroid cancer incidence in the USA in 2006 was 11,1/100.000 PE, corresponding to 33.100 newly detected cases (SEER Registry). This means that thyroid carcinomas were detected in 36% of thyroid surgeries - 4 times more frequently than in Germany.

**Table 2:** RAI recommendations in different guidelines / consensus papers.

	**RAI recommended**	**RAI considered**
German Association of Nuclear Medicine Guideline 2016 (6)	DTC > 1 cm N1 M1	DTC <1 cm: multifocality, capsule infiltration, infiltrative tumor growth, desmoplastic fibrosis, possibly BRAF V600E mutation, tumor diameter 6 - 10 mm, preoperative clinical carcinoma detection, history of familiarity, previous percutaneous irradiation of the soft tissues of the neck
European Association of Nuclear Medicine Guideline 2008 (7)	DTC > 1 cm DTC ≤ 1 cm without: - Unfavorable histology - History of radiation exposure - Evidence of metastasis - Thyroid capsule invasion	
British Thyroid Association Guideline 2016 (8)	pT3/4 M1 Any tumor with gross extra thyroidal extension	T1b/2 N1 with regard to: - Metastatic lymph nodes of large size - Multiple metastatic lymph nodes - Unfavorable histological type - Widely invasive histology - Large tumor size - Extrathyroidal extension - High ratio of positive to negative lymph nodes - Extracapsular nodal involvement
American Thyroid Association Guideline 2016 (4)	High-risk patients	Intermediate-risk patients (Low-risk patients with individual risk modifiers)
European Thyroid Association Consensus 2021 (3)	High-risk patients	Intermediate-risk patients (Low-risk patients with individual risk modifiers)

This difference makes it clear that the attention of diagnosticians and surgeons in the USA is much more focused on malignant changes in structural thyroid abnormalities than in Germany, where DTC is often discovered in the course of subtotal resection of an iodine-deficient goiter. This fact can be very well deduced from a large-scale international study of thyroid cancer self-help groups (12). The patients from Germany (*n* = 510) reported in 61% of the cases that they had to undergo completion surgery after the initial surgery. This percentage was half at 37% among patients from the United States (*n* = 919). The frequency of completion surgery in the Würzburg Thyroid Cancer Registry (15) is 56% (1114/1981), which is more comparable to the self-help study.

The consequent lack of oncological DTC resection results in a generally different exceptional situation in Germany with regard to further adequate tumor treatment.

## Evidence

The most relevant publications on this topic are the structured reviews and meta-analysis by Sawka et al., representing the inconsistency of existing studies regarding the effectiveness of RAI (16, 17). However, the tendency of improved outcomes after RAI, a statistically significant benefit in terms of reduction of recurrence rates and the rate of subsequent occurrence of distant metastases in patients with a tumor diameter >1 cm was shown in larger studies with a longer follow-up of more than 10 years. Highlighting the contradictions in literature indicates the fact that some studies find no benefit of RAI in non-metastasized mircocarcinomas while other authors find an improvement even in these cases (17–21).

Following the publication by Sawka et al. of the year 2008, in 2020 Verburg et al. published the latest review of the literature of the past decade (22). Included in this review and addressing RAI in intermediate-risk patients are the following publications.

21.870 patients with intermediate-risk (T1-3 N1 M0/x; T3 N0 M0/x) were studied by Ruel et al. from the United States National Cancer Database (NCDB) (23). These authors were able to show a significant improved overall survival, and in younger patients (<45 years) even a reduced risk of death after RAI.

8.061 patients from the Surveillance, Epidemiology, and End Results (SEER) Program database with an intermediate-risk (T1/2 N1; T3 N0/1) were analyzed separately (24). Zhang et al. were also able to show a significant improvement of RAI on the overall survival, however this improvement was not reproducible for disease specific survival.

In summary, there is credible evidence for the beneficial effects of RAI in intermediate-risk DTC patients, but strong evidence is lacking to provide definitive conclusions, thus controlled and randomized studies with long-term follow-up (>10 years) are urgently needed.

Currently, there are two studies whose aiming at this missing evidence, the ESTIMABL2 study and the IoN trial, both randomizing low- and intermediate-risk patients to either RAI or no RAI (25–27).

**Figure 1:**
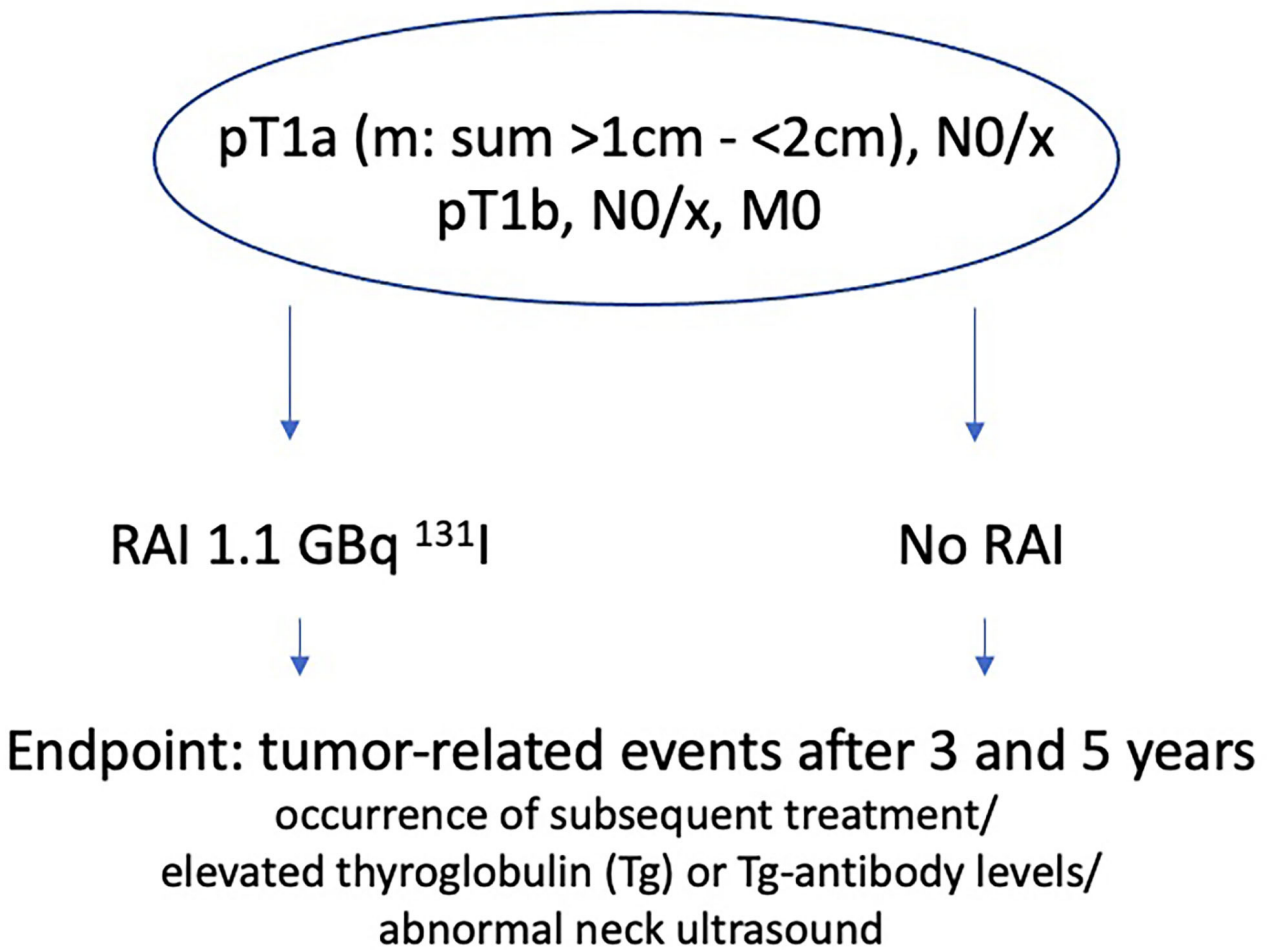
ESTIMABL2 study.

ESTIMABL2 thereby includes and randomizes patients of stage pT1a (m), N0/x, M0 with a sum of tumor size >1 cm and <2 cm or pT1b, N0/x, M0 (TNM 7th edition) who either get RAI with 1.1 GBq ^131^I or not, with the endpoint of tumor-related events after 3 and 5 years of follow-up (Figure 1). A tumor related event is defined by the occurrence of subsequent treatment (RAI administration or surgery) for abnormal RAI uptake on the post-therapeutic whole-body-scan (WBS) or by elevated thyroglobulin (Tg) or Tg-antibody levels and/or abnormal neck ultrasound during controls. The result of the 3 years follow-up in 726 French DTC patients just published, demonstrate non-inferiority of a follow-up strategy compared to systematic adjuvant post-operative of RIT [1.1 GBq ^131^I following recombinant human thyroid-stimulating hormone (rhTSH)] in low-risk DTC patients (26).

**Figure 2:**
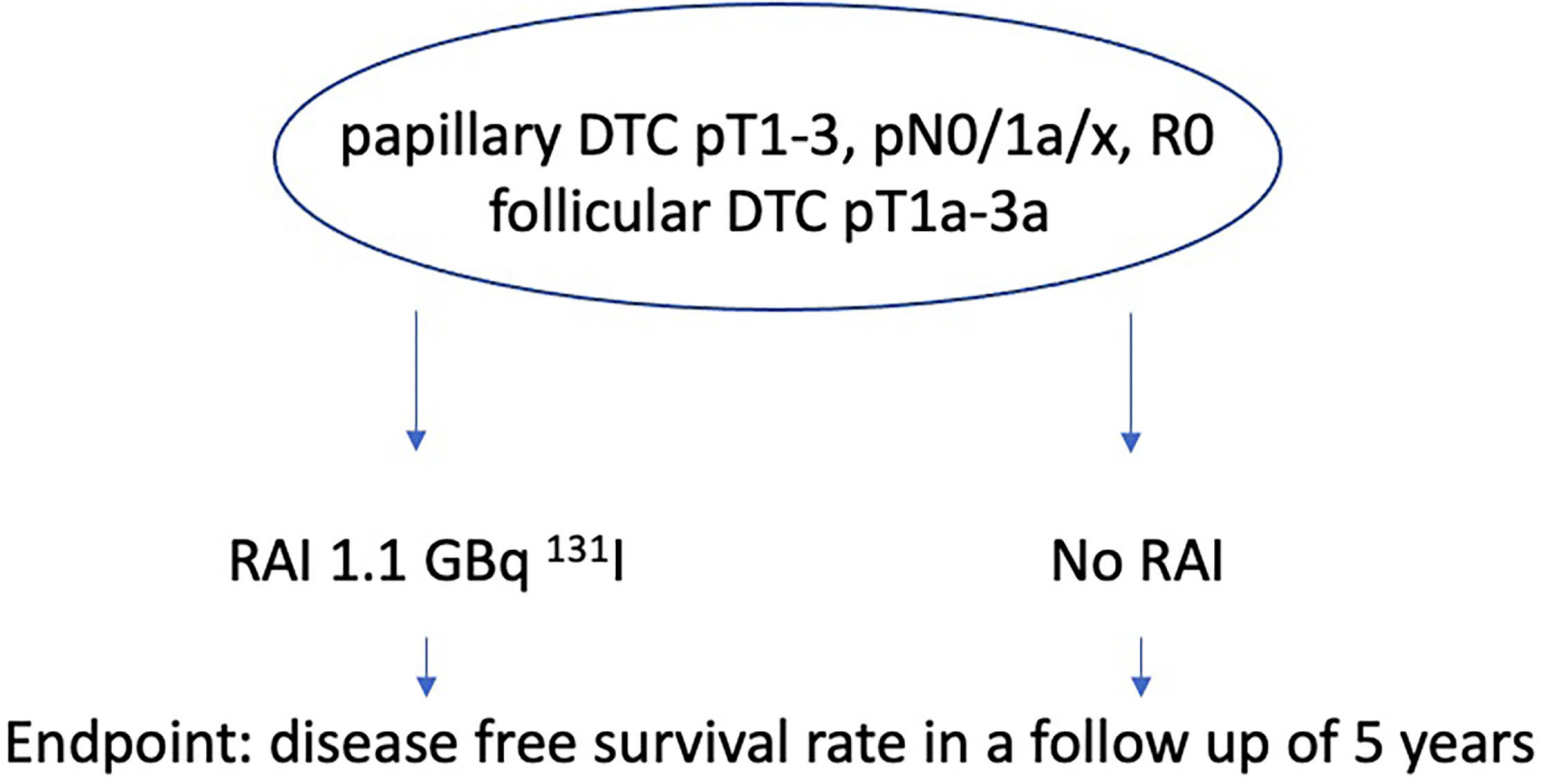
IoN study.

The inclusion criteria for the IoN study were defined as papillary DTC pT1-3, pN0/1a/x, R0, no aggressive histology, and follicular DTC pT1a-3a (TNM 8th edition) (Figure 2). Main objective is the disease free survival rate of patients receiving RAI with 1,1 GBq ^131^I and of those who don't in a follow-up of 5 years. This study started in 2011, the estimated primary completion date aiming at disease-free thyroid specific survival is 2023.

The short follow-up duration of the studies must be critically questioned, especially in view of the preceding literature, which was able to demonstrate the favorable effects of RAI after longer (>10 years) follow-up periods (16).

## Thyroglobulin, Ultrasound and Diagnostic Whole Body Scan to Refine RAI Therapy Decisions

The measurement of thyroglobulin and ultrasound of the neck are long established procedures to assess whether thyroid and/or tumor tissue is still present postoperatively, in the sense of a risk assessment. However, despite its establishment, this risk assessment is still limited. Campennì et al. demonstrated that in their studies collective, post therapeutic imaging revealed metastases in 82/570 (14.4%) patients, among them 77 cases with regional lymph node metastases only (28). The metastases were successively confirmed by histology or other diagnostic tools (sensitivity and PPV = 100%). 73/82 patients (90.2%) showed postoperative thyroglobulin levels ≤ 1 ng/ml, in 54% of the patients, thyroglobulin levels at RAI therapy remained ≤ 1 ng/ml. This work highlights that an undetectable / low measurable postoperative thyroglobulin value cannot be used with confidence to rule out metastasis.

In addition to the evaluation of Tg levels, Tg- antibodies should be evaluated. Elevated Tg-antibody levels might mask and interact with Tg values and should therefore be considered as a risk factor (29).

In a recent statement, the Martinique group advocates the additional use of pretherapeutic diagnostic whole body scans in selected patients in the intermediate-risk category (30). One argument for a diagnostic whole-body scan with ^131^I prior to therapy might be staging for example from an M0 to a M1 status which may lead to a change in the treatment plan. However, it should be emphasized that diagnostic ^131^I scans with low activity even including SPECT/CT imaging have lower sensitivity than post therapy scans, the limitations are also addressed by the group (30). Agate et al. showed in 545 low- and intermediate-risk patients, that less that 2% post-therapy scans showed distant metastases (31), which makes it obvious that only few patients may be “upgraded” by such imaging.

## Other Risk Factors

The heterogeneity of the patients included in the studies must be pointed out, as also mentioned by the Martinique group in their statement, additional possible risk factors are often not addressed (5). Moreover, many risk factors are only being discovered, are not yet well known, and are not well-established.

One possible risk factor is the BRAF (V600E) mutation. Several studies have shown a correlation between the presence of this mutation and a more aggressive course (32). Elisei et al. showed the BRAF (V600E) mutation as an independent poor prognostic factor for disease persistence in patients with low-risk DTC (33). So the presence of this mutation in patients should be considered in the decision to perform or not to perform radioiodine therapy.

In addition to the characteristics recorded in the ATA classification, other factors should be addressed. Among others, age should be discussed as a further risk factor, which is an important point for the classification of prognostic stage groups according to the American Joint Committee on Cancer (AJCC) / Union Internationale Contre le Cancer (UICC) (34). Zuhur et al. could show, that an age cutoff of 45 years may identify patients at a higher risk of persistence/recurrence in the ATA low- and intermediate-risk categories; besides disease-free survival was longer in females in the ATA low-risk category (35). Thus, sex is also a possible influencing factor. Another important issue seems to be the topography, Campennì et al. demonstrated the negative impact of tumor location in the thyroid isthmus on disease free survival and disease persistence 12 months after primary treatment (36).

## Importance of the Situation for Decision Making

Prospective randomized studies with long follow-up reliably assessing the benefit of RAI in intermediate-risk patients with regard to the reduction of the risk of recurrence and benefit in disease specific survival are lacking. There are many risk factors, some of which are not well known and established and we rely on dynamic risk stratifications (e.g. thyroglobulin). In view of this situation, participatory decision making between physicians and informed patients, which is in principle required today anyway, is of particular importance (5, 37). In the absence of strong evidence, the patient must be sufficiently informed about the evidence available and its background. So the patient can be actively involved in the choice of therapy in the sense of shared decision making.

The basis of this shared decision making is a strategy between physicians and patients in which the patient's values and preferences are combined with the best available medical evidence (38, 39). In this process, it is essential that the available evidence is fully displayed and explained by the physician and understood by the patient (39). In addition to the low level of evidence in this particular context, it has been shown that especially patients of female gender or educated patients with a University degree want to be actively involved in the decision making process, but it has been also demonstrated, that psychological distress may arise from this procedure (40).

For intermediate-risk DTC patients, the possible options are RAI as a therapeutic intervention or an “active surveillance” (or “wait-and-see”) approach. As reported by several studies in patients with prostate cancer (41), the decision to proceed with active surveillance may be accompanied by uncertainty and anxiety of the patient about the further course of the cancer that has not been “treated.” On the other hand, the decision for therapeutic intervention is understandably often associated with fears and anxieties about its risks and side effects, concerning radioiodine therapy especially with regard to secondary malignancies. It is essential that patients can fully engage in the decision-making process and feel confident in choosing treatment options (39).

D'Agostino et al. (42) were able to show that many patients with a known papillary microcarcinoma opted for surgery and against a wait-and-see approach in the context of shared decision making based on their personal needs and fears. From their perspective, surgery offered these patients the opportunity to resolve the uncertainty and emotional stress associated with their disease status and cancer diagnosis, they desired to have the cancer safely removed from their bodies and to return to a life they had lived before diagnosis. This study emphasizes how individual patients' needs, preferences and concerns were, concluding that detailed education about the available treatment options with their advantages and disadvantages is substantial. This situation can be transferred to intermediate-risk patients with the option of RAI. Similar results in the same context were published in a prospective study by Sawka and colleagues (43). Specifically, personal perceptions and concerns about physical or emotional well-being, as well as personal or family experiences related to cancer and surgery (including poor experience from own surgery and/ or death of a family member from advanced cancer), family considerations (including having a cancer parent in young families with children), timing of surgery in the context of life (including age, surgery as a limitation in terms of earning capacity), potential future risk (including fear of metastasis), and trust in treating physicians strongly influenced the decision between a wait-and-see approach and a therapeutic intervention. The included patients perceived choice as positive and were largely satisfied with their decision.

Davies et al. reported that 37% of their patients suffering from low-risk DTC under active surveillance would worry “sometimes” or “more often” because of the cancer, which was still to be found in 33% after 3 years (44). The main concerns related to tumor spread and growth. The colleagues conclude from their study that patients who opt for active surveillance worry to a similar extent as patients who undergo surgery. However, it remains an open question whether patients who made a conscious decision to opt for surgery would not have personally suffered more from these worries under active surveillance.

Hartzband and Groopman described patients as either being medical minimalists or medical maximalists (45). “Medical maximizers” are characterized by obtaining healthcare for even minor problems, as opposed to “medical minimizers,” who tend to avoid medical procedures that are not absolutely necessary (46). In a study by Tuttle et al. in DTC-patients (47), medical maximizers felt that surgery was necessary, feared the continued uncertainty of a wait-and-see approach, and saw surgery as the final tool for cure and control, many also expressed concern about possible metastases. The medical minimizers who opted for active surveillance viewed their disease as one of low-risk and were more likely to have concerns about thyroid hormone medications. In addition, according to the authors, it appeared that each patient processed the information, through personal perceptions and personal or family experiences, to reach a final decision.

Another interesting point from Tuttle's work (47) is the aspect that the authors believe that the physician's orientation toward his or her own personal medical decision making may consciously or unconsciously lead him or her to favor a more or less aggressive treatment option for low-risk thyroid cancer, such that physicians with a minimalist mentality tend to favor a less aggressive treatment approach, and physicians with a maximalist mentality tend to favor more aggressive treatment options. It is this presumption that all physicians should take to heart and try to adopt the attitude of a “neutral broker.”

The conclusions from studies in patients with the option of active surveillance or surgery for papillary microcarcinomas can be transferred, with a pinch of salt, to patients with intermediate-risk DTC with regard to shared decision making between a wait-and-see strategy or implementation of adjuvant radioiodine therapy. The focus of this process must be set on information provided by the physician as a “neutral broker.” The aim of this “empowerment” of the patients is to increase the degree of participation and autonomy, so that they can represent their ideas and preferences regarding treatment self-responsibly and self-determined in the sense of self-competence. The patient informed in this way should be enabled to make the decision based on his or her personal perception and preferences also in the absence of clear evidence for the superiority of one of the options.

## Conclusion

Recommendations for RAI in intermediate-risk patients differ between different countries and experts. Retrospective studies do not reach a clear conclusion, with a tendency of a benefit of RAI in large study groups with long follow-up periods. Prospective randomized studies assessing the benefit of RAI in intermediate-risk patients after sufficient long follow-up are lacking and comprehensive, indisputable evidence is likely to be lacking even after publication of recent studies. Thereby participatory decision making between informed patients and physicians is also of great interest in the longer term. Studies dealing with surgical treatment of papillary microcarcinomas can be tentatively applied to the situation of RAI in intermediate-risk patients. Furthermore, the focus should be on the patient who is fully informed by the physician and who makes the decision based on his/her own personality structure and experiences, in view of the presented evidence.

## Author Contributions

FE preparation of the manuscript. ML and CR manuscript improvement and finalization. All authors contributed to the article and approved the submitted version.

## Conflict of Interest

The authors declare that the research was conducted in the absence of any commercial or financial relationships that could be construed as a potential conflict of interest.

## Publisher's Note

All claims expressed in this article are solely those of the authors and do not necessarily represent those of their affiliated organizations, or those of the publisher, the editors and the reviewers. Any product that may be evaluated in this article, or claim that may be made by its manufacturer, is not guaranteed or endorsed by the publisher.
